# Isoaaptamine Induces T-47D Cells Apoptosis and Autophagy via Oxidative Stress

**DOI:** 10.3390/md16010018

**Published:** 2018-01-09

**Authors:** Chih-Fung Wu, Man-Gang Lee, Mohamed El-Shazly, Kuei-Hung Lai, Seng-Chung Ke, Chiang-Wen Su, Shou-Ping Shih, Ping-Jyun Sung, Ming-Chang Hong, Zhi-Hong Wen, Mei-Chin Lu

**Affiliations:** 1 Division of Surgical Oncology, Department of Surgery, Kaohsiung Medical University Hospital, Kaohsiung 807, Taiwan; hepatoma2003@yahoo.com.tw; 2 Division of Urology, Department of Surgery, Kaohsiung Armed Forces General Hospital, Kaohsiung 802, Taiwan; mg2253@yahoo.com.tw; 3 Department of Marine Biotechnology and Resources, National Sun Yat-sen University, Kaohsiung 804, Taiwan; 4 Division of Urology, Department of Surgery, Zuoying Branch of Kaohsiung Armed Forces General Hospital, Kaohsiung 813, Taiwan; ling92@ymail.com; 5 Department of Pharmacognosy and Natural Products Chemistry, Faculty of Pharmacy, Ain-Shams University, Organization of African Unity Street, Abassia, Cairo 115, Egypt; mohamed.elshazly@pharma.asu.edu.eg; 6 Department of Pharmaceutical Biology, Faculty of Pharmacy and Biotechnology, German University in Cairo, Cairo 114, Egypt; 7 National Museum of Marine Biology & Aquarium, Pingtung 944, Taiwan; mos19880822@gmail.com (K.-H.L.); mynameisr4@gmail.com (C.-W.S.); m6430005@hotmail.com (S.-P.S.); pjsung@nmmba.gov.tw (P.-J.S.); 8 Graduate Institute of Marine Biotechnology, National Dong Hwa University, Pingtung 944, Taiwan; 9 Department and Graduate Institute of Aquaculture, National Kaohsiung Marine University, Kaohsiung 811, Taiwan; junkrough.hmc@webmail.nkmu.edu.tw

**Keywords:** anti-cancer, apoptosis, autophagy, isoaaptamine, Nrf2/p62, ROS

## Abstract

*Aaptos* is a genus of marine sponge which belongs to Suberitidae and is distributed in tropical and subtropical oceans. Bioactivity-guided fractionation of *Aaptos* sp. methanolic extract resulted in the isolation of aaptamine, demethyloxyaaptamine, and isoaaptamine. The cytotoxic activity of the isolated compounds was evaluated revealing that isoaaptamine exhibited potent cytotoxic activity against breast cancer T-47D cells. In a concentration-dependent manner, isoaaptamine inhibited the growth of T-47D cells as indicated by short-(MTT) and long-term (colony formation) anti-proliferative assays. The cytotoxic effect of isoaaptamine was mediated through apoptosis as indicated by DNA ladder formation, caspase-7 activation, XIAP inhibition and PARP cleavage. Transmission electron microscopy and flow cytometric analysis using acridine orange dye indicated that isoaaptamine treatment could induce T-47D cells autophagy. Immunoblot assays demonstrated that isoaaptamine treatment significantly activated autophagy marker proteins such as type II LC-3. In addition, isoaaptamine treatment enhanced the activation of DNA damage (γH2AX) and ER stress-related proteins (IRE1 α and BiP). Moreover, the use of isoaaptamine resulted in a significant increase in the generation of reactive oxygen species (ROS) as well as in the disruption of mitochondrial membrane potential (MMP). The pretreatment of T-47D cells with an ROS scavenger, *N*-acetyl-l-cysteine (NAC), attenuated the apoptosis and MMP disruption induced by isoaaptamine up to 90%, and these effects were mediated by the disruption of nuclear factor erythroid 2-related factor 2 (Nrf 2)/p62 pathway. Taken together, these findings suggested that the cytotoxic effect of isoaaptamine is associated with the induction of apoptosis and autophagy through oxidative stress. Our data indicated that isoaaptamine represents an interesting drug lead in the war against breast cancer.

## 1. Introduction

Studies on natural products have demonstrated that their unique scaffolds can assist plants, marine organisms and microorganisms to survive and thrive in their natural habitats [1,2,3]. Researchers understood the biological impact of secondary metabolites and used them as a backbone for the development of therapeutic agents against many ailments. The last century witnessed breakthroughs in understanding the nature of many diseases and how to treat them. However, certain territories remained unconquered and despite four decades since the declaration of war on cancer, this disease remains one of the major threats to human well-being [4]. Fortunately, human beings were not totally defeated in their war on cancer and certain impressive accomplishments were achieved in the form of drugs targeting and halting the progress of this debilitating disease. Natural products played a significant role in such accomplishments through targeting proteins necessary for cancer cell survival and replication [5]. Terrestrial plants and marine organisms provided a huge library of anticancer agents such as alkaloids, terpenoids, and flavonoids [6]. Out of the 27,000 different alkaloids derived from plants, more than 17,000 demonstrated diversified pharmacological properties including anticancer activities [7]. Plant-derived alkaloids exhibit promising cytotoxicity against many cancer cells through the induction of DNA damage, activation of caspases and inhibition of cell growth [7,8]. 

The marine environment, which remains scarcely investigated for its therapeutic agents, comprises a wide array of organisms from seaweeds to sponges living in harmony under harsh environmental conditions. With the introduction of new techniques of samples collection, many biologically active molecules were discovered from marine organisms [9]. It is estimated that at least two-thirds of novel chemical structures isolated between 2010 and 2012 from marine sponges exhibited potent cytotoxicity against a panel of cancer cell lines. The isolated compounds fell into four main chemical classes including terpenoids, alkaloids, macrolides, and peptides, which along with polyketides, and sterols showed a wide range of biological activities [10]. In the last decade, our group has focused on the identification of cytotoxic agents from marine organisms through studying their effect and molecular mechanisms of action using in vitro and in vivo models aiming to find a potent cytotoxic drug lead [11,12]. In a continuation of our work, we examined the content of *Aaptos* sp. methanolic extract which led to the isolation of known spongean aaptamines alkaloids, aaptamine (Ap), isoaaptamine (IAp) and demethyloxyaaptamine (DAp). Previous studies indicated that aaptamine and its congeners exhibited potential interesting biological effects including anti-HIV, antifungal, anti-photoaging, anti-infective, antifouling, antidepressant, antiviral, antimalarial, and cytotoxic activities [13,14,15,16,17,18,19]. Since the isolation of Ap in 1982 by Nakamura et al., most of the work done on these alkaloids focused on the identification of their biological activities without revealing the molecular mode of action. Only recently, aaptamines alkaloids were found to target proteins in certain cancer lines. Proteomic-based screening approaches suggested that aaptamines alkaloids demonstrated potent cytotoxic effect by targeting myc, p53 and TNF molecules in cisplatin-sensitive and -resistant germ tumor cells [20,21]. However, aaptamines alkaloids cytotoxic mechanism of action against other cancer cell lines remains elusive.

In the past few decades, numerous studies revealed the role of ROS (reactive oxygen species) in tumorigenesis and anticancer therapy [22]. ROS are actively produced by cancer cells to stimulate survival, proliferation, differentiation, metastasis, invasion, and angiogenesis of cancer cells by inducing and maintaining oncogenic phenotypes [23,24,25,26]. On the other hand, accumulating evidence implicated a relationship between the increase in the intracellular levels of ROS and the induction of apoptosis by most conventional chemotherapeutic drugs through the promotion of DNA damage and activation of NFκB and AP-1 of cancer cells [27,28,29]. Therefore, it was concluded that ROS exhibit a dual role in fighting tumorigenesis and cancer. Several reports indicated that nuclear factor erythroid 2-related factor 2 (Nrf 2), a transcription factor, which mainly regulates cellular defenses against oxidative stress and electrophilic insults by up-regulating the expression of various detoxifying/antioxidant genes, including NADPH quinine oxidoreductase, heme oxygenase-1, glutathione generation enzymes, and GSH peroxidase as well as drug efflux transporters [30,31,32]. The induction of Nrf2 is activated by p62 which accumulates in the cytosol to competitively bind with keap 1 and translocates into the nucleus to transactivate the antioxidant target genes, such as heme oxygenase 1 (HO-1), NAD(P):quinone oxidoreductase (NQO1), glutathione S-transferase (GST) and γ-glutamyl cysteine synthetase catalytic subunit (GCLc) [32,33,34,35]. Emerging evidence suggested that p62 is a hub of multiple signaling pathways in HER2-induced mammary tumorigenesis via multiple cellular proliferation- and survival-related signaling pathways, including PI3K/Akt and canonical WNT/β catenin [33,36,37].

In this study, we sought to elucidate the cytotoxic activity of aaptamine alkaloids in vitro along with their mechanism of action. The effect of IAp treatment on ROS generation and ER stress-related proteins in breast T-47D cancer cells was evaluated showing an enhancement in ROS generation as well as IRE 1α and Bip expression. Based on the results that ROS and ER stress are involved in IAp effects on T-47D cells, we hypothesized that the induction of apoptosis and autophagy might be attributed to mitochondrial dysfunction-dependent apoptosis and Nrf2/p62 dependent autophagy.

## 2. Results 

### 2.1. Isoaaptamine Isolated from Sponge Aaptos sp. Induces Apoptosis and Autophagy in Breast T-47D Cancer Cells 

**Figure 1:**
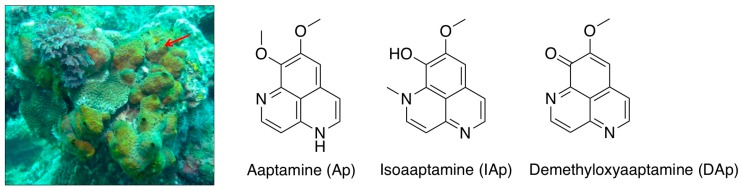
Morphology of marine sponge *Aaptos* sp. collected from the coast of Ping-Tung in 2012 and the three major active alkaloids.

**Figure 2:**
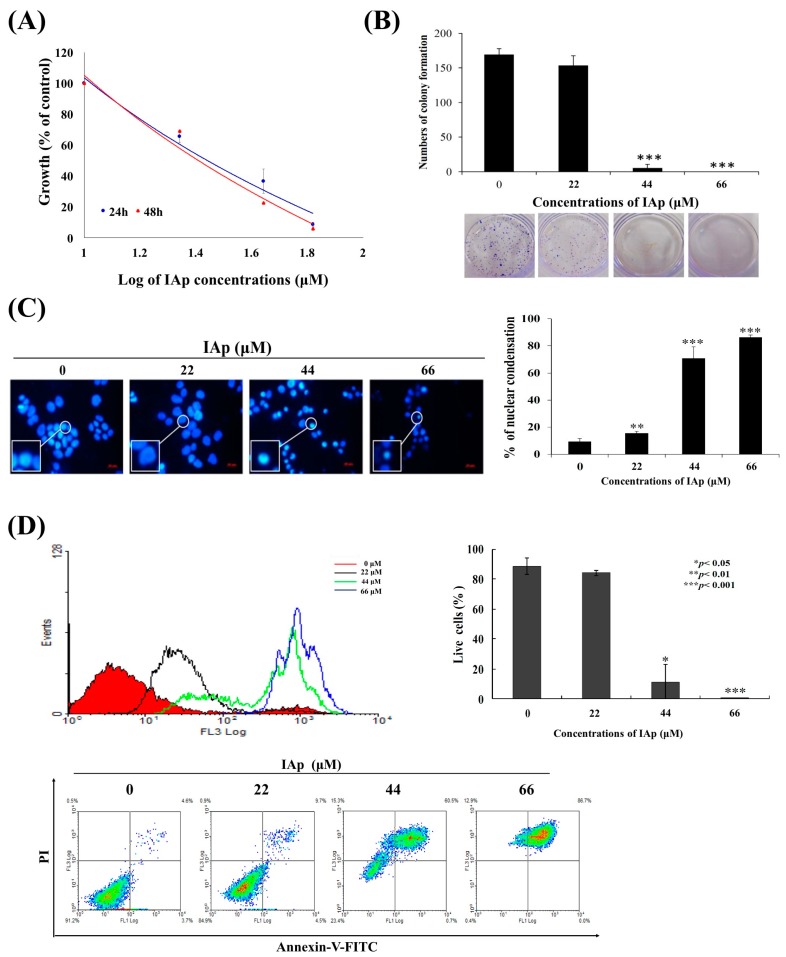

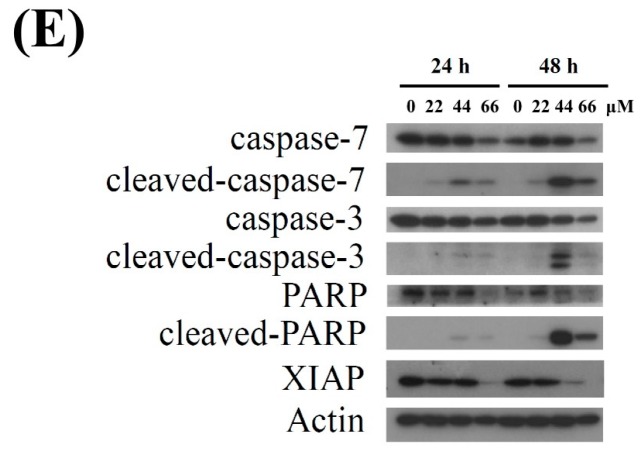
IAp suppresses cells growth and induces apoptosis in human breast cancer T-47D cells. Cells were treated with different concentrations of IAp for 24 and 48 h, respectively. (**A**) Cells growth was determined by the MTT assay. (**B**) IAp inhibited colony formation of T-47D. Cells grown in six-well plates (700 cells/well) were treated with the indicated concentrations of IAp for 6 h, and then changed with fresh medium without any drug treatment for 14 days. Formed colonies were stained and counted as described in the “Methods section”. Data are expressed as the mean ± SD of three experiments. (* *p* < 0.05; ** *p* < 0.01; *** *p* < 0.001 compared with the control groups). Cells were treated with the indicated concentrations of IAp for 24 h and (**C**) stained with DAPI and morphological changes were examined and counted by fluorescent microscopy. Data are expressed as the mean ± SD of three experiments. (* *p* < 0.05; ** *p* < 0.01; *** *p* < 0.001 compared with the control groups); (**D**) they were also stained with annexin V/PI and examined using flow cytometric assay; (**E**) and the expression of apoptotic-related proteins was determined with Western blotting assay. Actin was the loading control.

Cytotoxic constituents of the sponge *Aaptos* sp. (Figure 1) were isolated using bioactivity-guided fractionation, including the previously known spongean aaptamine alkaloids, aaptamine (Ap), isoaaptamine (IAp) and demethyloxyaaptamine (DAp). The cytotoxic effect of the three major constituents was evaluated. MTT assay was used to evaluate the effect of these compounds on different human breast cancer cell lines, including MCF-7, MDA-MB-231 and T-47D cells (Table 1). Ap was not active against all breast cancer cells at 88 μM for 72 h treatment. DAp exhibited the most potent cytotoxic activity with an IC_50_ of 23.11 ± 2.36 μM, 19.34 ± 3.77 μM and 33.02 ± 8.49 μM against MCF-7, MDA-MB-231 and T-47D cells. The IC_50_ values of IAp against MCF-7, MDA-MB-231 and T-47D cells were 49.12 ± 12.28, 49.56 ± 2.19, and 30.13 ± 3.07 μM. Although DAp was the most potent cytotoxic agent against all cancer cell lines, we decided to work on IAp because it was the most prominent alkaloid (84.74%) in the active fraction and showed comparable activity to DAp. IAp exhibited the most potent activity against T-47D cell line suggesting its potential as a growth inhibitory agent against breast cancer cells. These results prompted a further investigation to reveal IAp cytotoxic mode of action against T-47D cells. We further determined the anti-proliferative effect of IAp (0, 22, 44 and 66 μM) on T-47D cells with MTT assay for 24 h and 48 h, respectively. As shown in Figure 2A, IAp at concentrations of 22, 44 and 66 μM significantly suppressed cell growth in a concentration- and time-dependent manner. The short-term antiproliferative effect of IAp as demonstrated by MTT assay was further confirmed by the long-term antiproliferative (colony formation) assay. T-47D cells were diluted and seeded into six-well plates at a density of 700 cells/well. After IAp treatment, colonies were stained with violet crystal and counted (>50 cells) under a microscope. After 14 days of incubation, the clonogenic formation ability of T-47D cells was examined. IAp suppressed colonies formation compared with the solvent control in a concentration-dependent manner (Figure 2B). To assess the nuclear morphological changes induced by IAp, T-47D cells were stained with 4′,6-diamidino-2-phenylindole (DAPI) and examined under a fluorescence microscope. IAp treatment significantly increased the percentage of condensed nuclei about 15.6%, 70.9% and 86.3% compared with the control, which showed intact and normal nuclei (Figure 2C).

Recent studies confirmed the link between the potential application of secondary metabolites as cytotoxic agents and their apoptotic- and autophagic-inducing effects [38,39]. Alkaloids were among these secondary metabolites which exhibited cytotoxic activity against human carcinoma cells through the induction of apoptosis and autophagy [40,41]. To better comprehend the cytotoxic mechanism of IAp, the population of apoptotic cells was determined using annexin V/PI assay. As indicated in Figure 2D, after 24 h of treatment, the population percentage of apoptotic cells (annexin-V and PI-positive) was significantly increased by 16.03%, 88.77% and 99.77% compared with the negative control. We then attempted to identify the precise mechanism by which IAp mediates apoptotic cell death of T-47D cells. The hallmarks of classical apoptosis (cleavage of Poly (ADP-ribose) polymerase (PARP) and caspase 7 as well as the expression of X-linked inhibitor of apoptosis protein (XIAP)) were determined by Western blotting analysis. We treated T-47D cells with different concentrations of IAp for 24 and 48 h. IAp treatment caused a concentration-dependent activation of caspase 3, caspase 7 and cleavage of PARP, which confirmed the induction of apoptosis (Figure 2E). IAp inhibited the expression of XIAP, a caspase inhibitor, in a concentration- and time-dependent manner, suggesting that IAp treatment activated caspase pathway via the inhibition of XIAP expression.

**Figure 3:**
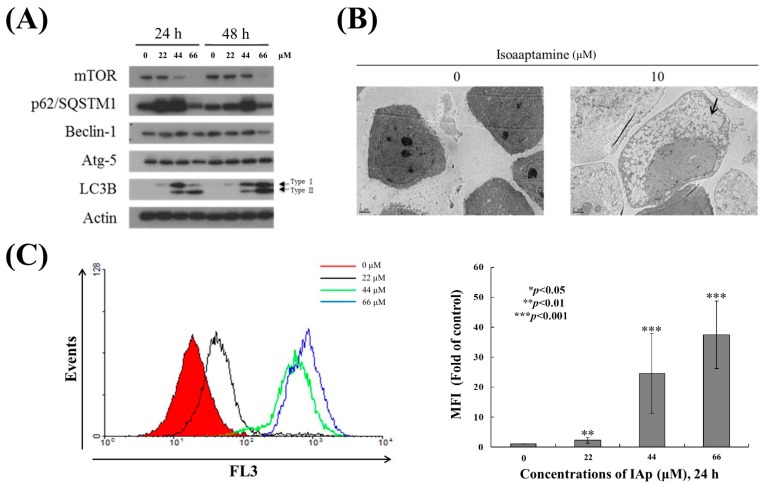
IAp induced autophagic hallmarks in T-47D cells. (**A**) Effect of IAp on the expression of autophagy-related proteins. Cells were treated with the indicated concentrations of IAp for 24 h and 48 h. Western blotting analysis was performed with mTOR, p62/SQSTM1, Beclin 1, Atg 5, and LC3B antibodies. Actin was the loading control. (**B**) Cells were treated with 44 μM of IAp for 24 h. Images of TEM were examined after treatment. (**C**) T-47D cells were treated with the indicated concentrations of IAp for 24 h. After treatment, cells were incubated with acridine orange for 30 min at 37 °C and analyzed using flow cytometry. Quantitative analysis of proton-pumping V-type ATPase activity showed a gradual increase of red fluorescent intensity upon IAp treatment when compared with the control group. Data are expressed as the mean ± SD of three experiments (* *p* < 0.05; ** *p* < 0.01; *** *p* < 0.001 compared with the control groups).

We then moved to identify the precise mechanism by which IAp mediates autophagic cell death of T-47D cells. The hallmarks of classical autophagy (activation of LC 3B II and expression of mTOR and p62/SQSTM1) were determined by Western blotting analysis. We treated T-47D cells with different concentrations of IAp for 24 and 48 h. In agreement with a previous study, IAp treatment at 22 and 44 μM resulted in a concentration-dependent upregulation of LC3-II (autophagosome marker) and accumulation of p62/SQSTM1 (autophagy-related marker) after 24 h, which indicated autophagic flux (Figure 3A) [42]. In addition, IAp treatment suppressed the expression of mTOR, the downstream effector of PI3K/Akt pathway [43] in a concentration- and time-dependent manner, suggesting that IAp treatment elicited the accumulation of p62/SQSTM1 via suppressing mTOR expression in agreement with a previous report [44]. We used transmission electron microscopy (TEM), which is one of the most sensitive techniques to monitor autophagy [45], to examine the intracellular morphological changes of T-47D following 24 h of IAp treatment (44 μM). As shown in the electron micrographs, cells treated with IAp demonstrated an increase of autophagic vacuoles compared with the control group (Figure 3B). We further used 3 μM of acridine orange (AO) as a probe to determine the lysosomal activity of T-47D cells treated with IAp for 24 h. The data showed that the mean fluorescent intensity of T-47D cells treated with 22, 44 and 66 μM of IAp increased 2.09 ± 0.9, 24.5 ± 13.3, and 37.3 ± 11.2 folds, respectively compared with the control group (Figure 3C), suggesting the uptake of AO and its accumulation in acidic vesicles [46].

**Figure 4:**
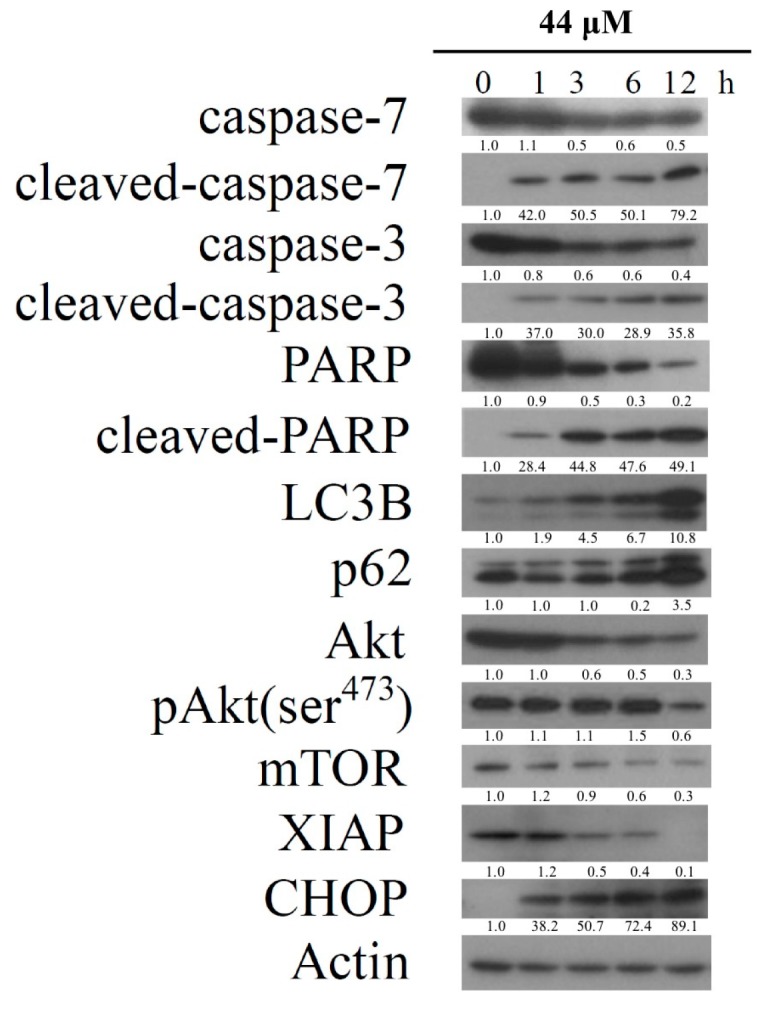
IAp induced the hallmarks of apoptosis and autophagy in T-47D cells. Effect of IAp on the expression of apoptotic-, autophagy- and prosurvival-related proteins. Cells were treated with 44 μM of IAp for the indicated time intervals. Western blotting analysis was performed with these specific antibodies. Actin was the loading control.

We studied a time course response of IAp on the levels of apoptotic-related proteins, cleaved caspases-3, -7 and PARP; autophagy-related proteins, p62 and LC3-II; and prosurvival enzymes XIAP, p-Akt and mTOR. The levels of apoptotic-related proteins including cleaved caspases-3, -7, and PARP by IAp treatment (44 μM) were increased 35.77 ± 2.28, 52.71 ± 14.98 and 7.96 ± 1.80 folds, respectively compared with the levels of the negative control after 12 h. IAp also up-regulated the levels of p62 and LC3-II 3.52 ± 0.72 and 10.78 ± 2.60 folds, respectively compared with the levels of the negative control after 12 h. Furthermore, IAp exposure decreased the levels of p-Akt (ser^473^), mTOR and XIAP as well as diminished the level of CHOP, which resulted in IAp-mediated apoptosis and autophagy (Figure 4). We also found that the long-term exposure of IAp promoted the induction of apoptosis and autophagy after 24 h and 48 h (Figure 2 and Figure 3).

### 2.2. Effect of IAp on the Disruption of Mitochondrial Membrane Potential and the Expression of Mitochondrial Glycolysis-Related Proteins in Breast T-47D Cancer Cells 

**Figure 5:**
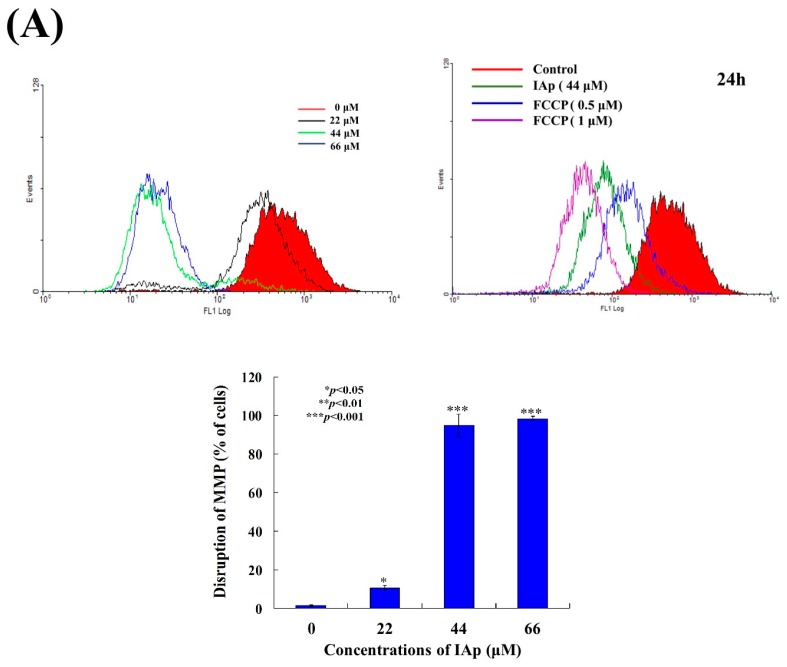

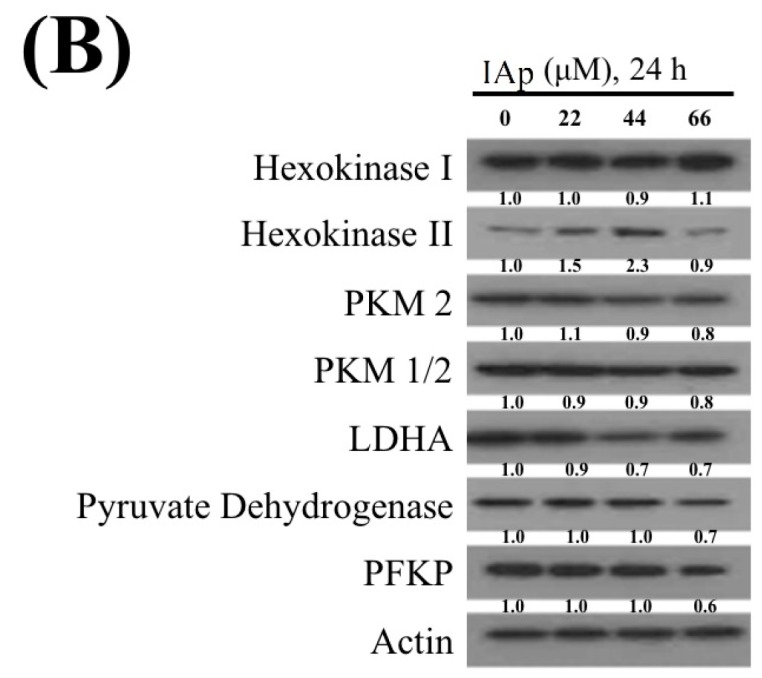
Effect of IAp on the disruption of mitochondrial membrane potential and expression of mitochondria-related proteins. (**A**) Cells were treated with different concentrations of IAp (0, 22, 44 and 66 μM) for 24 h. Quantitative results of the fluorescent intensity of mitochondrial membrane potential showed a gradual increase in the MMP disruption upon the treatment with IAp when compared with the negative/positive control groups. Results are presented as mean ± SD of three independent experiments. (**B**) Expression of mitochondrial glycolysis-related proteins was determined by Western blotting assay. Cells were treated with different concentrations of isoaaptamine for 24 h. Western blot analysis was performed with hexokinase I and 2, PKM1/2 and 2, LDHA, pyruvate dehydrogenase, and PFKP antibodies. Actin was used as an internal control to show the equal loading of the proteins.

To address whether the induction of apoptosis by IAp was related to the mitochondrial pathway, rhodamine 123 fluorescent dye was used to determine changes in mitochondrial membrane potential (MMP). T-47D cells were treated with different concentrations of IAp for 24 h and then stained with rhodamine 123. As shown in Figure 5A, the use of IAp (22 μM) increased the population of T-47D cells with disrupted membrane potential from 1.56% to 10.65%. This effect was dramatically increased with the treatment of IAp at 44 and 66 μM, resulting in 94.88% and 98.37% cells with disturbed MMP, respectively (Figure 5A). To further understand the mechanism of IAp-induced MMP disruption, the effect of IAp on the proteins related to mitochondrial metabolism was evaluated. As shown in Figure 5B, IAp treatment did not change the expression of hexokinase I and PKM1/2. Taken together, IAp treatment diminished hexokinase II, pyruvate dehydrogenase, PFKP, and PKM2, but enhanced the expression of hexokinase II.

### 2.3. Effect of IAp on ROS Generation and the Expression of Endoplasmic Reticulum (ER) Stress-Related Proteins in Breast T-47D Cancer Cells 

**Figure 6:**
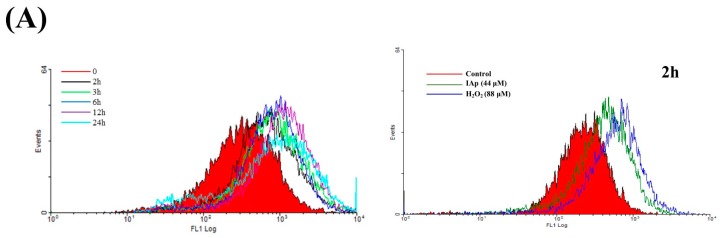

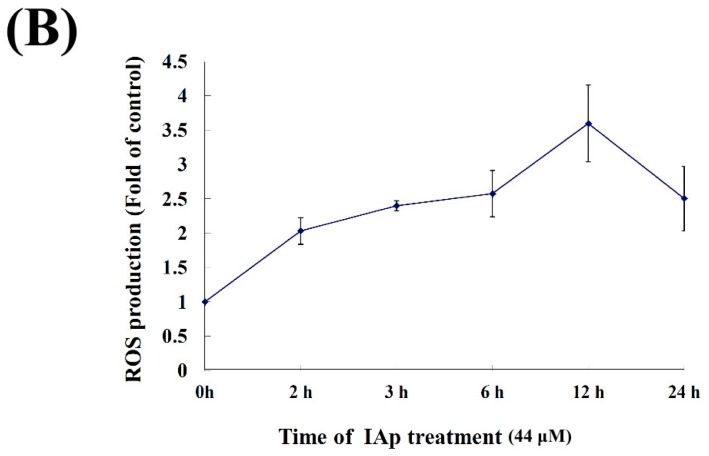
Effect of IAp on reactive oxygen species (ROS) generation in T-47D cells. Cells were treated with 44 µM of IAp for the indicated time intervals and analyzed by flow cytometry. (**A**) Histogram profiles of the negative/positive controls and drug treatments that were measured by flow cytometry. (**B**) Quantitative analysis of the changes in ROS level showed a gradual increase in the ROS production upon IAp treatment when compared with the control group. Results are presented as mean ± SD of three independent experiments.

Reactive oxygen species (ROS) produced by mitochondria or external environmental factors affecting cultured cells have been implicated as a signal of autophagosome formation and the induction of autophagy [47]. The induction of the intracellular formation of ROS by IAp was determined with a carboxyl derivative of fluorescein, carboxy-H_2_DCFDA dye using flow cytometric analysis [12]. The levels of ROS at different time intervals following IAp treatment were determined to examine whether the IAp-induced apoptosis and autophagy in T-47D cells involve the overproduction of ROS. A carboxy derivative of a fluorescein dye, carboxy-H_2_DCFDA, was used to examine a time-dependent increase in ROS generation. IAp treatment (44 μM) for 2, 3, 6, 12, and 24 h resulted in 2.03 ± 0.19, 2.40 ± 0.07, 2.58 ± 0.34, 3.59 ± 0.56 and 2.50 ± 0.47-folds increase in the ROS levels, respectively, in comparison with the mean fluorescence index (MFI) of the control (Figure 6).

**Figure 7:**
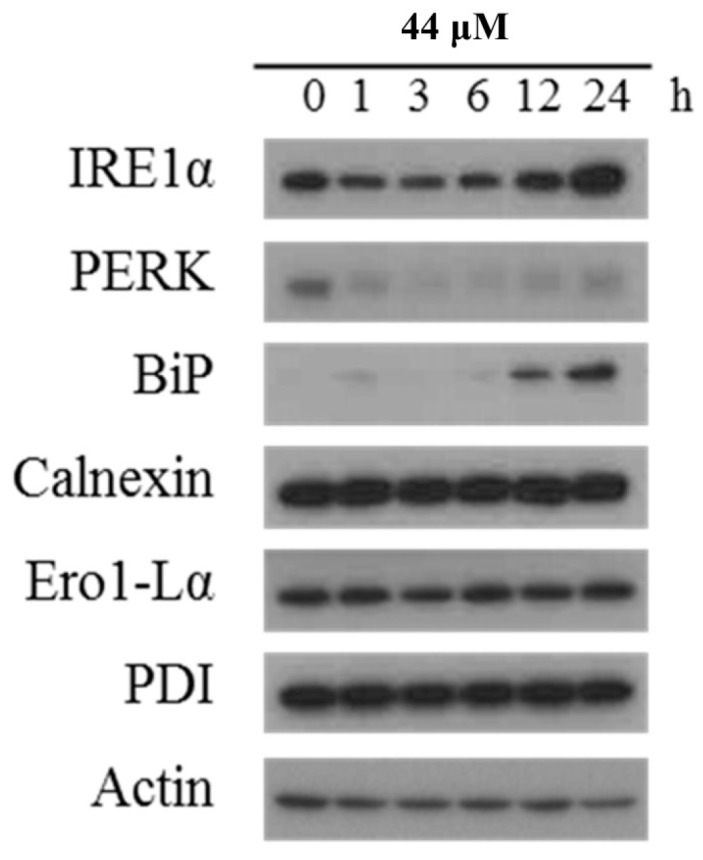
Effect of IAp on endoplasmic-reticulum (ER) stress-related proteins. T-47D cells were treated with 44 μM of IAp for the indicated time intervals. Western blotting analysis was performed with IRE 1α, PERK, BiP, calnexin, Erol-Lα and PDI antibodies. Actin was used as an internal control to show the equal loading of the proteins.

In addition, ROS generation can induce ER stress leading to mitochondrial-related apoptosis or autophagy [48,49]. To further investigate if ER stress is involved in the apoptotic and autophagic effect induced by IAp, Western blotting analysis was used to determine the expression of ER stress-related proteins. In a time-dependent manner, IAp promoted the levels of binding immunoglobulin protein (Bip) and inositol-requiring enzyme 1α (IRE 1α) but suppressed the levels of protein kinase R (PKR)-like endoplasmic reticulum kinase (PERK) (Figure 7).

### 2.4. Apoptosis and Autophagy Induced by IAp Is Mediated by Excessive ROS Generation

**Figure 8:**
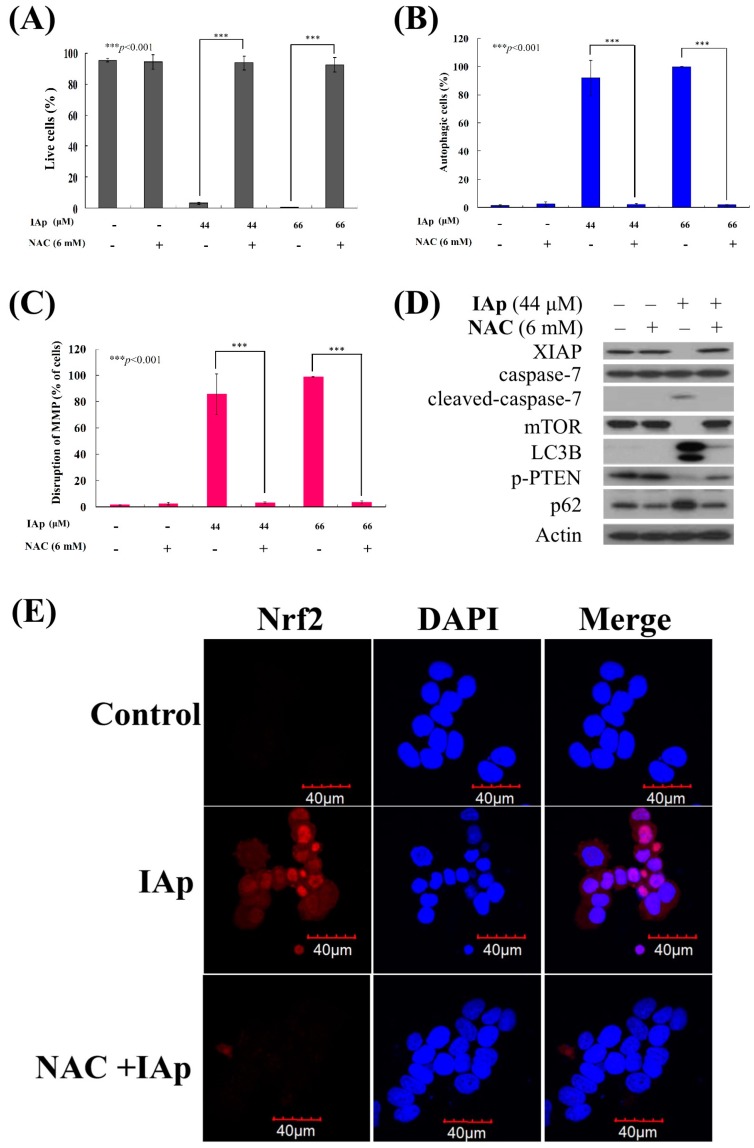

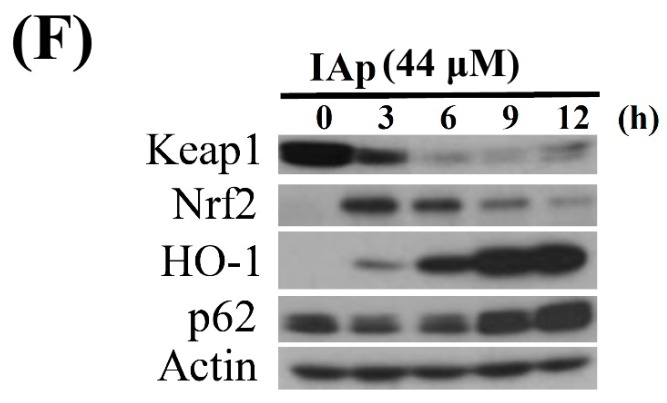
Effect of NAC on apoptosis- and autophagy-induced by IAp treatment. Cells were pretreated with NAC (6 mM) for 2 h and were further treated with 44 and 66 μM of IAp for 24 h. The living population (**A**); the autophagic population (**B**); and the disruption of MMP (**C**) were examined with annexin-V/PI, acridine orange and rhodamine 123 staining using flow cytometric analysis. Results shown are the mean ± SD of three independent experiment (*** *p* < 0.001); (**D**) Western blotting analysis was performed with XIAP, cleaved-caspase 7, p62, LC3B and p-PTEN antibodies. Actin was used as an internal control to show the equal loading of the proteins; (**E**) Effect of NAC on the translocation of Nrf2 by IAp treatment in T-47D cells using immunofluorescence by confocal microscope; (**F**) Effect of IAp on the expression of antioxidant Keap1–Nrf2 pathway with Western blotting assays. Actin was used as an internal control to show the equal loading of the proteins.

We wanted to investigate if ROS generation is involved in IAp-induced apoptosis and autophagy. To achieve this goal, T-47D cells were pretreated with 6 mM *N*-acetyl-l-cysteine (NAC), an ROS scavenging agent, aiming to counteract the intracellular oxidative stress. Apoptotic cells population was determined via annexin V/PI staining after treatment with NAC. Cells treated with NAC demonstrated similar staining pattern to the negative control group, showing less than 5% of apoptotic cells population (Figure 8A). In addition, the pretreatment with 6 mM NAC completely blocked the apoptotic and autophagic cell population from 96.2% and 99.99% to 3.8% and 7.2% as well as 91.87% and 99.96% to 2.24% and 1.74% in response to the use of 44 and 66 μM of IAp, respectively. These findings suggested that blocking the oxidative stress by NAC suppressed apoptosis and autophagy induced by IAp (Figure 8A,B). We further examined the relationship between the disruption in MMP induced by IAp and ROS overproduction through evaluating the effect of NAC pretreatment on the population of T-47D cells with disturbed MMP. Rhodamine 123, a cationic dye, was used to determine the population of T-47D with disturbed MMP (Figure 8C). According to the experimental design, T47D cells were divided into four groups, in which two groups were only treated with IAp (44 or 66 μM), and the other two groups were treated with NAC (6 mM) followed by IAp (44 or 66 μM). The changes in cells population with disturbed MMP was examined after 24 h. The population of cells with disturbed MMP significantly declined in response to NAC pretreatment from 85.78% and 98.95% to 3.09% and 3.18%, respectively. The effect of NAC pretreatment on the expression of the apoptotic and autophagy-related proteins was also evaluated. NAC pretreatment abrogated the suppression of mTOR, XIAP and PTEN phosphorylation as well as the induction of Nrf2 expression induced by IAp using Western blotting and immunocytofluorescence analysis (Figure 8D,E). It also suppressed the activation of caspase 7, LC 3B, and p62. Taken together, the cytotoxic effect of IAp against T-47D cells is mediated through apoptotic and autophagic induction, as well as mitochondrial dysfunction and ER stress involving ROS over-generation. To explore if the Nrf2/keap 1 signal pathway involved in the cytotoxic activity of IAp, the expression of keap 1 was significantly decreased by IAp treatment from 3 h to 12 h, accompanied by an increase of cytosolic Nrf2 content. Following the treatment of T-47D cells with IAp for various times, significant increase in heme oxygenase (HO-1) and p62/SQSTM1(p62) was observed using Western blotting analysis (Figure 8F).

## 3. Discussion

Aaptamine was the first member to be isolated from a group of alkaloids which came to be known as aaptamines [50]. They are characterized by the presence of a benzo[de][1,6]naphthyridine ring in their framework. All aaptamines were obtained from *Demospongiae*, a class of marine sponges, (also called “horny sponges” or “siliceous sponges”), the largest class in the phylum Porifera. Since their isolation, they attracted a lot of attention due to their unique structures and potent biological activities. They exhibited anti-HIV, anti-fungal, anti-photoaging, anti-infective, anti-fouling, anti-depressant, anti-viral, anti-malarial, and cytotoxic activities [13,14,15,16,17,18,19]. Even a bronchodilator drug, benafentrine, has been developed on the basis of benzo[c][1,6]naphthyridines nucleus and acted as a phosphodiesterase III/IV inhibitor [51].

Several reports emphasized the cytotoxic effect of aaptamine analogs through the modulation of AP-1, NFκB and p53-dependent transcriptional activity in mouse JB6 Cl41 cells and cisplatin-resistant germ cancer cells [20,21]. In this study, we found that the major active alkaloid, IAp, demonstrated potent cytotoxicity against several breast cancer cell lines, T-47D, MCF-7 and MDA-MB-231 with promising IC_50_ values (Table 1) and induced autophagy and apoptosis of T-47D cells (Figure 2 and Figure 3). Our results indicated that IAp induced cytotoxic activity in T-47D cells through interrupting the transcription of ER stress-related proteins, induction of mitochondrial dysfunction and stimulation of ROS overexpression (Figure 8). The elucidated mechanism may contribute to the development of IAp as an anti-cancer drug candidate.

Apoptosis and autophagy are well-known mechanisms underlying cell death induced by anticancer compounds, such as quercetin, metformin, diosmin, and polyphyllin GA [52,53,54,55]. Our results indicated that IAp treatment inhibited T-47D cell growth and colony formation in a time- and concentration-dependent manner. The compound suppressed the growth of T-47D cells by the induction of apoptosis and autophagy. The apoptosis-induced by IAp was characterized by chromatin condensation, phosphatidylserine externalization, and cleavage of caspase 7 and PARP as well as suppression of XIAP, which is a specific inhibitor of caspases after 24 and 48 h (Figure 2). Simultaneously, evidences of autophagy induced by IAp were demonstrated by the activation of LC 3 type II, an increase of p62/SQSTM1 expression and the formation of autophagy vacuoles, a decrease in mTOR expression and elevation of AO^+^-cell population after 24 and 48 h (Figure 3). A previous study confirmed that metformin, a well-known anti-diabetic drug, could promote apoptosis of hepatocellular carcinoma through a CEBPD-induced autophagy pathway [53].

In this study, we delineated the cytotoxic effect of IAp against T-47D cells, which was manifested by the induction of apoptosis and autophagy. It was also revealed that ROS generation was involved in the process. Our results indicated that p62/SQSTM1 played a fundamental role in IAp-induced apoptosis and autophagy in T-47D cells (Figure 8D). Previous studies demonstrated that p62/SQSTM1 is a stress-inducible cellular protein that possesses multiple domains. This ubiquitin-binding adaptor or scaffold protein mediates its interactions with various binding partners such as a signaling hub for mammalian targets of rapamycin complex 1 (mTORC1) activation on lysosomes and the Keap1–Nrf2 pathway on autophagic cargos, as well as an adaptor/receptor for selective autophagy [33]. It was found that p62/SQSTM1 promotes Her2-induced mammary tumorigenesis through multiple signaling pathways, including the PTEN-PI3K/Akt, and Nrf1/Keap1 axis. It was also demonstrated that the overexpression of Her2/Neu resulted in PTEN downregulation via the absence of p62 [56]. Recent results demonstrated that electrophiles and oxidants switch on Nrf2-dependent cellular defense mechanism resulting in Nrf2is release from Keap1 and translocation into nucleus to conserve the antioxidant response element sequence for the balance of redox homeostasis [57,58]. It was reported that Keap 1 was modified via oxidative-dependent mechanism, which might be sequestered by p62 as a scaffold for several protein aggregates triggering their degradation through proteasome or lysosome pathways via autophagy [59]. Furthermore, p62, which is a direct Nrf2-targeted gene, increased the expression of Nrf2 by binding to ARE sequence in the p62 promoter [60]. However, Nrf2-Keap1 homeostasis is as a new and unique mode of nuclear–cytoplasmic collaboration by controlling the cellular response not only to oxidative and xenobiotic stresses but also potentially to stress induced by mechanical injury via NF-κB/I-κB or microtubule-based signal transduction [61,62]. Nikolaos et al. described that a novel Nrf2 inducer, HB229/PMI, increased the cellular expression of p62 by reversibly inhibiting the regulatory activity of Keap 1. Additionally, the inducer disrupts protein–protein interaction, thereby blocking the ubiquitination of Nrf 2 and promoting its nuclear accumulation [58,63]. In a recent study, cell exposure to IAp treatment did not enhance the nuclear accumulation of Nrf2, whereas notably depleted the expression of Keap 1 (Figure 8F). Whether the activation of p62 disrupts the Nrf1/Keap1-mediated antioxidant mechanism remains to be investigated. Additionally, previous studies indicated that the silencing of p62 suppressed ROS generation, suggesting that p62 accumulation is related to ROS generation [64,65,66,67]. The current study showed that IAp induced T-47D cell apoptosis and autophagy via cellular Nrf1/Keap1 antioxidant depletion and ROS accumulation (Figure 6B and Figure 8D,E). These effects were abrogated with the pretreatment of NAC, which is a ROS scavenger. Certain reports suggested that the induction of autophagy is reduced with p62 expression. In our work, we found that the induction of autophagy by IAp is related to p62 accumulation. Our findings were consistent with a previous study on a traditional herbal medicine, SH003, which suppressed breast cancer growth by inducing autophagy through promoting p62/SQSTM1 (Figure 8D) [68]. Our results provide compelling evidence on the regulatory role of p62/SQSTM1 in ROS-dependent cell death aiming to aid in the development of IAp into a clinical drug.

## 4. Experimental Section 

### 4.1. Bioassay Materials

American Type Culture Collection (ATCC, Manassas, VA, USA) was the source for all cell lines. Cell lines were kept at 37 °C in a humidified atmosphere of 5% CO_2_ in RPMI 1640 medium supplemented with 10% fetal calf serum, 2 mM glutamine and antibiotics (100 units/mL of penicillin and 100 μg/mL of streptomycin). Trypan blue, fetal calf serum (FCS), RPMI 1640 medium, streptomycin, and penicillin G were purchased from GibcoBRL (Gaithersburg, MD, USA). 3-(4,5-Dimethylthiazol-2-yl)-2,5-diphenyl-tetrazolium bromide (MTT), dimethyl sulfoxide (DMSO), p62 and all other chemicals were obtained from Sigma-Aldrich (St. Louis, MO, USA). Antibodies against mTOR, Beclin-1, actin, LC3B, c-caspase 3 and 7, c-PARP, PKM1/2, Atg-5, pKM2, pyruvate dehydrogenase, LDHA, PFKP, p-PTEN (Ser^380^), PERK, IRE-1α, Calnexin, Bip, PDI, and Ero1-Lα were acquired from Cell Signaling Technologies (Beverly, MA, USA). Antibodies for Chop, XIAP, p-Akt (Ser^473^), Nrf2, hexokinase I and II and Keap1 were purchased from Santa Cruz Biotechnology (Santa Cruz, CA, USA). Carboxy derivative of fluorescein (carboxy-H_2_DCFDA) and rhodamine 123 cationic dye were obtained from Molecular Probes and Invitrogen Technologies (Carlsbad, CA, USA). Anti-mouse and rabbit IgG peroxidase-conjugated secondary antibody were acquired from Pierce (Rockford, IL, USA). Annexin V-FITC/PI (propidium iodide) stain was obtained from Strong Biotech Corporation (Taipei, Taiwan). Hybond ECL transfer membrane and ECL Western blotting detection kits were purchased from Amersham Life Sci. (Amersham, UK).

### 4.2. Preparation of the Marine Alkaloid Stock Solution

Aaptamines were isolated from *Aaptos* sp. and their chemical structures were elucidated by analyzing their spectroscopic data (1D and 2D NMR) and comparing those data to a previous report [68]. Each compound was dissolved in DMSO (20 μg/μL) and diluted before use.

### 4.3. MTT Proliferation Assay

Culture plates (96-well) were used in the MTT assay. Cells were seeded at 4 × 10^4^ per well and then treated with different concentrations of the tested compounds [69]. The cytotoxic effect of the tested compound was determined by MTT cell proliferation assay (thiazolyl blue tetrazolium bromide, Sigma-M2128) for 24, 48 or 72 h. ELISA reader (Anthoslabtec Instrument, Salzburg, Austria) was used to measure light absorbance values (OD = OD_570_ − OD_620_) at 570 and 620 nm. The concentration that caused 50% inhibition (IC_50_) was calculated. These results were expressed as a percentage of the control ± SD established from *n* = 4 wells per experiment from three independent experiments.

### 4.4. Annexin V/PI Apoptotic Assay

Phosphatidylserine (PS) externalization and membrane integrity were measured utilizing annexin V-FITC staining kit [69]. Cells (10^6^) were grown in 35-mm diameter plates and were labeled with annexin V-FITC (10 μg/mL) and PI (20 μg/mL) before to harvesting. All plates were washed after labeling with a binding buffer and then harvested. The binding buffer was used to resuspend cells at a concentration of 2 × 10^5^ cells/mL before assessment on an FACS-Caliburflow cytometer (Beckman Coulter, Taipei, Taiwan) and analyzed with CellQuest software. Approximately 10,000 cells were counted for each measurement.

### 4.5. Determination of ROS Generation and MMP Disruption

Determination of ROS Generation and MMP Disruption were performed as described previously [69]. MMP disruption and ROS generation were examined with rhodamine 123 cationic dye (5 μg/mL) and the carboxy derivative of fluorescein (carboxy-H_2_DCFDA, 1.0 mM), respectively. Cells treated with the tested compounds were labeled with a specific fluorescent dye for 30 min. Cells were washed with PBS after labeling and resuspended in PBS at a concentration of 1 × 10^6^ cells/mL before analysis using flow cytometry.

### 4.6. Immunofluorescence Analysis

The treated-cells were fixed with 4% paraformaldehyde in 50 mM HEPES buffer (pH 7.3) for 30 min after treatment with the tested compound. Cells were then permeabilized for 20 min with 0.2% Trition X-100 in PBS (pH 7.4). Cells were incubated with 5% BSA in PBS containing 0.05% Trition X-100 (T-PBS) for 1 h at room temperature to prevent non-specific protein binding. Incubation of the cells was done with the primary Nrf2 antibodies (1:250) for 2 h followed by secondary antibodies (Alexa Fluor 586-conjugated goat anti-mouse IgG (H + L)) (Life Technologies, Carlsbad, CA, USA) diluted at 1:1000 for 1 h at room temperature. Cells were washed with PBS and observed under a FV1000 confocal laser scanning microscope (Olympus, Tokyo, Japan).

### 4.7. Statistics

Results were expressed as the mean ± standard deviation (SD). An unpaired Student’s *t*-test, was used to compare each experiment and a *p*-value of less than 0.05 was considered to be statistically significant.

## 5. Conclusions

Our study showed that spongean alkaloids, aaptamine (Ap), isoaaptamine (IAp) and demethyloxyaaptamine (DAp) exhibited potent cytotoxicity against breast cancer cells including MCF-7, T-47D and NDA-MB-231 cells. The most abundant component in the active fraction, IAp, inhibited T-47D growth via autophagy-mediated apoptosis. Our study deciphered that IAp induced apoptosis and autophagy through p62-dependent oxidative stress in breast cancer T-47D cells. These findings add a further piece to the jigsaw puzzle of IAp mode of action against breast cancer cell lines which will assist scientists in their attempt to develop this alkaloid into a therapeutic agent.

## Figures and Tables

**Table 1:** Cytotoxicity of marine alkaloids isolated from sponge *Aaptos* sp. against several breast cancer cells for 72 h (IC_50_ μM).

	T-47D	MCF-7	MDA-MB-231
Aaptamine (Ap)	NA *^a^*	NA *^a^*	NA *^a^*
Isoaaptamine (IAp)	30.13 ± 3.07	49.12 ± 12.28	49.56 ± 2.19
Demethyloxyaaptamine (DAp)	33.02 ± 8.49	23.11 ± 2.36	19.34 ± 3.77
Staurosporine *^b^*	0.45 ± 0.01	0.11 ± 0.01	0.45 ± 0.01

*^a^* NA, not active at 88 μM; *^b^* Positive control.
